# A Day at the Rural District Hospital: What Can We Learn about Healthcare Quality?

**DOI:** 10.4269/ajtmh.21-0500

**Published:** 2021-07-16

**Authors:** David Gaus, Diego Herrera

**Affiliations:** Andean Health and Development, Brisas de Colorado, Sector 1, Santo Domingo de los Tsachilas, Ecuador

5:30 AM, Roosters cackling, time to wake up:

I walk the half kilometer to the district 20-bed hospital as the sun is rising. Three hours from the capital through mountainous terrain, the hospital serves a three-county agricultural community of 70,000 inhabitants, most living in poverty. It supports five public health outpatient facilities and several smaller social security health outposts, thus straddling the public and private sector. I am the hospital’s only full-time attending physician today, working with medical residents and part-time specialists.

6:30 AM, Morning report:

The first patient presented is a 60-year-old woman with diabetes, hypertension, and heart failure who is unable to get her medications regularly because the healthcare system frequently experiences shortages. She’s in heart failure again. The on-call resident then presents the other 14 indigent patients. Their conditions include pneumonia, dengue, stroke, myocardial infarction (MI), diarrhea/dehydration, pyelonephritis, motor vehicle accident, complicated childbirth, venomous snakebite, appendicitis, cholecystitis, and a suicide attempt. I notice the resident’s documentation in the medical record displays major deficiencies, principally because his medical school training and 2 years in a public government outpatient center did not emphasize written documentation. He pays attention to my feedback, but I know—just like the one before him and the one before that—he really just wants to return to the capital to find a job there.

8:00 AM, Bedside rounds with the patients, nurse, and resident physicians:

The team informs me that snake antivenom is not available, and the suppliers don’t know when they will replenish it. Without it, the boy would require a trip to the capital—a journey he likely would not survive. The woman with pyelonephritis is on her fourth hospital day and remains febrile in spite of broadening antibiotic coverage, which I still apply blindly because cultures are not available to determine bacterial sensitivity. The young man with the motor vehicle accident suffered a femur fracture and closed head trauma. I have been waiting 2 days for the orthopedic surgeon to come from a neighboring province to set the fracture and am unsure if the patient needs a CAT scan of his brain to rule out a bleed. The man with the stroke cannot get out of bed, he is malnourished, there are no rehabilitation services available, and I know that sending him home will likely result in his rapid demise.

9:00 AM Rounds continue:

The laboring mother is 35 weeks pregnant with pre-eclampsia. I can induce her delivery today, but I’m not sure if the newborn will need more than just the usual resuscitation. There are no neonatal ICU beds available at the referral hospital in the capital. The man with the MI appears to be stable, but he develops chest pain while I examine him. All the right medications have been prescribed for his acute condition, but the family is asking about heart catherization, which I know is unlikely as only a few interventional cardiology laboratories exist in the country. Our part-time general surgeon for the patient with appendicitis is available, but the anesthesiologist is currently on vacation, and his covering partner is not answering my calls. Finally, I am very concerned that our suicidal patient, if sent home, would attempt suicide again. There are no psychologists or psychiatrists anywhere in our regional primary care system.

9:30 AM, Electricity challenges:

The appendectomy is about to begin when the power grid goes down. Per protocol, the maintenance person attempts to start the backup generator, but because budgets have tightened, no allocations were made for fuel. The hospital general administrator was able to scrape up some cash to buy the fuel needed for the backup generator. The appendectomy finally proceeds.

9:45 AM, Managing worsening chest pain:

The man with the MI develops worsening chest pain, so I have a medical resident call a fellow resident at the referral hospital; without this contact and personal outreach, I know that the chances of successful transfer are slim. Furthermore, the electronic heart monitor is now damaged since the power outage produced an initial power surge, so I am unable to follow electrocardiogram changes in this patient.

10:00 AM, confronting supply shortages:

The orthopedic surgeon finally comes to set the femur fracture but discovers that the screws and plate ordered by the hospital are, in fact, the wrong size. He leaves the hospital without discussing the situation with me, leaving a brief message to call when the proper supplies arrive. I discover that the hospital is late on paying the provider of orthopedic supplies. They won’t deliver more supplies unless a payment has been made. The patient’s mentation is still altered, and I must decide if it is bad enough to put the patient and his family through the burden of finding a CAT scanner for further evaluation.

10:15 AM, The complicated childbirth proceeds:

I discuss delivery options with the mother with pre-eclampsia, and together the decision is made to proceed with the delivery without transfer. She is nervous because her previous labor resulted in the death of her newborn at our hospital 2 years ago when my colleague mismanaged a shoulder dystocia. Labor proceeds uneventfully; the newborn is slightly depressed but responds positively to usual resuscitation measures. I breathe a sigh of relief.

10:20 AM, Outpatient continue waiting:

Our 40 patients in the outpatient clinic grow frustrated as we continue to be pulled away for the hospitalized and emergency patients. Many of them suffer from diabetes and other chronic, noncommunicable diseases that should be managed at the primary care level, but the general primary care physicians scattered around the hospital catchment area have little experience with cardiovascular pathology and therefore refer care to the hospital. I have proposed workshops and training for those physicians, but the regional ministry of health office has focused its administrative efforts and resources on other disease-specific vertical programs, such as tuberculosis, malaria, dengue, and the questionable threat of seasonal influenza.

3:00 PM, Morning clinic ends:

I finally finish morning clinic with the residents, but several patients had to leave unattended, as their only transportation to their remote, small communities leaves at 1:30 PM.

3:45 PM, Discussion with family:

After much discussion, the family of the stroke patient tells me that they want to take him home. They believe that a shaman cast a spell on him, and they need to find another shaman for a cleansing. I express sympathetic understanding but am ultimately able to convince them to keep him in the hospital until he regains some strength.

4:00 PM, Mental health and medical decision-making:

I have limited mental health training as a family physician and know that I am in over my head with the suicidal young man. I connect with the primary care physician from the patient’s community to discuss options, but she regrets that she cannot offer any formal therapy interventions. There is nowhere else to turn, and the family is looking to me for help. I decide to keep the patient another day, hoping to offer enough support to help him avoid another suicide attempt.

4:30 PM, Transport troubles:

Word arrives that the referral hospital has agreed to receive the patient with the MI. The ambulance begins its perilous 3-hour journey up the mountains, only to break down after 1 hour. No maintenance had been performed on the ambulance in 9 months, and the engine overheated.

6:00 PM, Walking home and reflecting:

I finish afternoon rounds with the team, complete medical records, and do some teaching to the residents and nurses. My goal with education is to inspire learners, to help them to locally contextualize the biomedical model imported from unfamiliar settings, and to be critical of their own healthcare system to be better advocates for their patients.

On my walk back home, I remind myself of the very positive impact the hospital has on the health of the community, but I return to the same systemic reflections that nag me every day.

Our hospital is a non-government organization district hospital that works with the local public sector primary care network and the social security system in rural Ecuador. This account, written in a pre-pandemic world, cannot capture all conditions in the wide range of rural district hospitals in low- and middle-income countries, public or private, but it can highlight many of the challenges these hospitals face. Much global health policy has been generated around universal coverage, yet the quality of that coverage seems to attract scant attention. Our hospital uses evidence-based treatment protocols. In fact, we write evidence-based medical manuals. Yet, there are so many other factors when considering quality of care. How that quality is measured is the task of others, but I would suggest that, in addition to typical quantitative, statistical analysis, the world should know the goings on in small rural hospitals and the infrequently considered challenges we face, particularly in rural areas of low- and middle-income countries with struggling healthcare systems.

From my perspective after 25 years in this hospital, the root of many of the health sector problems that rural district hospitals are forced to confront revolves around political and economic power, corruption, and incompetence, reflecting the greater society. In fact, attempts at health sector reform seem to reduce a profound, complex structural and systemic problem into merely an organizational problem, which explains repeated reform failure. Rather, an analysis of “quality of care” seems to require greater complexity and depends on multiple factors such as geography, economics, and the social position and political power of families, among other cultural factors. But it starts with seeing what actually happens to real people experiencing their health system.

